# Linking Migraine to Gut Dysbiosis and Chronic Non-Communicable Diseases

**DOI:** 10.3390/nu15204327

**Published:** 2023-10-11

**Authors:** Manuela Di Lauro, Cristina Guerriero, Kevin Cornali, Maria Albanese, Micaela Costacurta, Nicola Biagio Mercuri, Nicola Di Daniele, Annalisa Noce

**Affiliations:** 1 Department of Systems Medicine, University of Rome Tor Vergata, 00133 Rome, RM, Italy; manuela.dilauro@ptvonline.it (M.D.L.); cristinaguerriero@hotmail.it (C.G.); cornali.kevin@hotmail.it (K.C.); maria.albanese@hotmail.it (M.A.); mercurin@med.uniroma2.it (N.B.M.); didaniele@med.uniroma2.it (N.D.D.); 2 Neurology Unit, Headache Center, Tor Vergata University Hospital, 00133 Rome, RM, Italy; 3 Department of Surgical Sciences, University of Rome Tor Vergata, 00133 Rome, RM, Italy; micaela.costacurta@uniroma2.it; 4 Fondazione Leonardo per le Scienze Mediche Onlus, Policlinico Abano, 35031 Abano Terme, PD, Italy; 5 UOSD Nephrology and Dialysis, Policlinico Tor Vergata, 00133 Rome, RM, Italy

**Keywords:** migraine, chronic non-communicable diseases, gut microbiota, nutritional approaches, lifestyle changes

## Abstract

In the world, migraine is one of the most common causes of disability in adults. To date, there is no a single cause for this disorder, but rather a set of physio-pathogenic triggers in combination with a genetic predisposition. Among the factors related to migraine onset, a crucial role seems to be played by gut dysbiosis. In fact, it has been demonstrated how the intestine is able to modulate the central nervous system activities, through the gut–brain axis, and how gut dysbiosis can influence neurological pathologies, including migraine attacks. In this context, in addition to conventional pharmacological treatments for migraine, attention has been paid to an adjuvant therapeutic strategy based on different nutritional approaches and lifestyle changes able to positively modulate the gut microbiota composition. In fact, the restoration of the balance between the different gut bacterial species, the reconstruction of the gut barrier integrity, and the control of the release of gut-derived inflammatory neuropeptides, obtained through specific nutritional patterns and lifestyle changes, represent a possible beneficial additive therapy for many migraine subtypes. Herein, this review explores the bi-directional correlation between migraine and the main chronic non-communicable diseases, such as diabetes mellitus, arterial hypertension, obesity, cancer, and chronic kidney diseases, whose link is represented by gut dysbiosis.

## 1. Introduction

Migraine is a complex neurological disorder that triggers a particular type of headache, characterized by unilateral, pulsating, and moderate–severe pain, which generally worsens with physical activity and is associated with other symptoms, such as nausea, vomiting, and photophonophobia [1]. With an estimated global prevalence of 14.7% [2], the World Health Organization (WHO) listed migraine among the top ten causes of disabilities worldwide [3]; it is in first place if we consider people younger than 50 years old [4]. Women are 3–4 times more affected than men and show more disabling and drug-resistant, attacks and this consequently provokes a great socio-economic burden [5,6,7]. About 3% of the patients, suffering from episodic migraine, evolves every year towards a more complex clinical picture of chronic migraine. In this latter condition, patients should suffer a monthly migraine of about 15 or more days, in the last three months [8].

**Figure 1:**
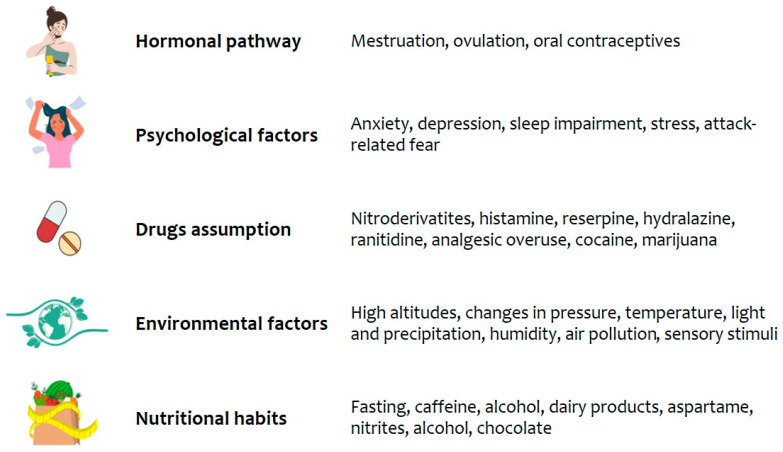
Common trigger factors for migraine development and chronicization.

The main trigger factors of this migraine progression are hormonal pathways, psychological syndromes, drugs assumption, environmental factors, and nutritional habits (Figure 1).

In women, migraine can be linked to the fluctuations in sexual reproductive hormones. In fact, decreased estrogens in the late luteal phase of the menstrual cycle lead to an increased permeability of blood vessels to prostaglandins, becoming a migraine trigger [9]. On the contrary, elevated estrogen levels are associated with more frequent pain attacks, especially with aura [10]. For this reason and for a higher stroke risk, associated with aura, the use of oral contraceptives is still restricted by the current guidelines in those women affected by this headache subtype [11]. The most frequently experienced psychological factors associated with this pathological condition are (i) anxiety, which seems to have a shared genetic basis with a migraine [12,13]; (ii) depression [12,14], characterized by a low availability of 5-hydroxytryptamine (5-HT), an increased sensitivity of trigeminovascular pathways [15], the hypofunction of the dopaminergic system [14], a down-regulation of the GABAergic system, and decreased estrogen levels; (iii) stress [16], namely, previous and recurrent stressful events that have been shown to be correlated with migraine onset and its chronicization [17]; and (iv) an excessive fear of migraine attacks, which refers to the fear of a headache occurring and that can worsen the disease course [18]. Even the use of some psychoactive drugs can be an important provoking agent for migraine generation, including nitroderivates, histamine, reserpine, hydralazine, and ranitidine, as well as cocaine and marijuana [19,20,21,22,23].

Notably, the overuse of analgesic or other abortive pain medications (i.e., triptans) is the major clinical factor of a migraine worsening, inducing a particular condition, known as “medication overuse headache” [24]. Among the environmental factors that can lead to the migraine development are high altitudes, changes in the atmospheric pressure, temperature, light and precipitation, humidity, air pollution, and sensory stimuli, such as olfactory and visual ones [25]. Impaired sleep patterns can also promote migraine attacks, particularly in young adults [26]. Other important migraine triggers are nutritional habits and certain foods, including fasting, caffeine, natural sweeteners (such as aspartame), nitrites of preserved meats, biogenic monoamines of alcohol, chocolate and dairy products (such as cheese and yogurt), and monosodium glutamate [25].

Migraine is frequently associated with chronic non-communicable diseases (CNCDs), such as diabetes mellitus (DM), arterial hypertension (AH), obesity, and cancer and chronic kidney disease (CKD). Moreover, CNCDs are themselves both the cause and consequence of a negative change in the gut microbiota composition, called “dysbiosis” [27,28,29].

In this regard, in addition to pharmacological treatments, a new adjuvant therapeutic strategy able to counteract migraine can be represented by different nutritional approaches able to modulate the gut microbiota composition and gut–brain axis [30].

The term “microbiota” means a very wide community consisting of bacteria, viruses, fungi, Archea, and unicellular eukaryotes. The term “microbiome”, instead, indicates a set of genetic patrimonies of microorganisms, which constitute it [31].

The microbial component and its genetic patrimonies are affected by lifestyle, dietetic habits, and other external factors, such as the environment. Therefore, the gut microbiota is a dynamic system that is in constant evolution [32] and is also composed by the vascular gut barrier [33]. The latter is a fundamental coating system able to control epithelium permeability and the passage of potentially pathogenic molecules and bacteria into the bloodstream [34,35]. Therefore, the microbiota can be defined as a “meta-organ”, namely, it is a structure that anatomically is not a part of the organism; however, it accompanies human phylogenetic evolution [36,37].

The aim of this narrative review is to analyze the possible correlation between migraine and gut microbiota dysbiosis and the possible relationship between migraine and CNCDs. Moreover, we examine the possible nutritional approaches and lifestyle changes able to positively modulate gut microbiota composition, reducing migraine frequency and intensity.

## 2. Materials and Methods

In order to achieve the review’s aim, a literature search was conducted using three databases (PubMed, Scopus, and Cochrane Library), until August 2023. The search was limited to peer-reviewed journals, written in the English language, and the search terms were “migraine” in combination with “gut microbiota”, AND “chronic degenerative non communicable diseases”, AND “diabetes mellitus”, AND “Arterial Hypertension”, AND “obesity”, AND “cancer”, AND “chronic kidney disease”, AND “Mediterranean Diet”, AND “Ketogenic Diet”, AND “probiotics”, AND “physical exercises”, AND “vitamins”, AND “iron”, AND “polyphenols”, AND “electrolytes”, AND “histamine”.

## 3. The Gut Microbiota Physiological Composition

The first studies related to the discovery of the microbiota date back to the beginning of the twentieth century, when a student of Pasteur, Elie Metchnikov [38] explored the theme of symbiotic bacteria in organisms, unlike Pasteur who focused his attention mainly on pathogenic ones. Metchinikov described the beneficial effects of lactic ferments on the bacterial flora and health host [38]. Subsequent studies have pointed out the presence of these bacteria that are involved in the absorption of nutrients as well as in immune system modulation [39,40]. In the 1990s, for the first time, the “gut–brain axis” was highlighted, namely, how the microbiota exerted a physiological role in the maintenance of host health [41].

The human microbiota is characterized by a high bacterial density, with a metagenome that in the adult is 150-times larger than the overall human genetic pattern [42]. It is an ecosystem composed by a set of ecological niches in close contact with the intestinal mucosa, forming an area of 250–400 m^2^ [37]. A more precise view of the complexity of the gut microbiota has been made in recent years, thanks to the metagenomic data.

Although there is a wide variety of symbiotic microorganisms, among the 160 species present in the human intestine, of which at least 57 are common to all individuals, the most widespread microbial phyla are only 4: Bacteroides, Firmicutes, Actinobacteri, and Proteobacteria. The first two are present in a higher quantity; in fact, they constitute about 90% of the human gut microbiota [43,44].

**Figure 2:**
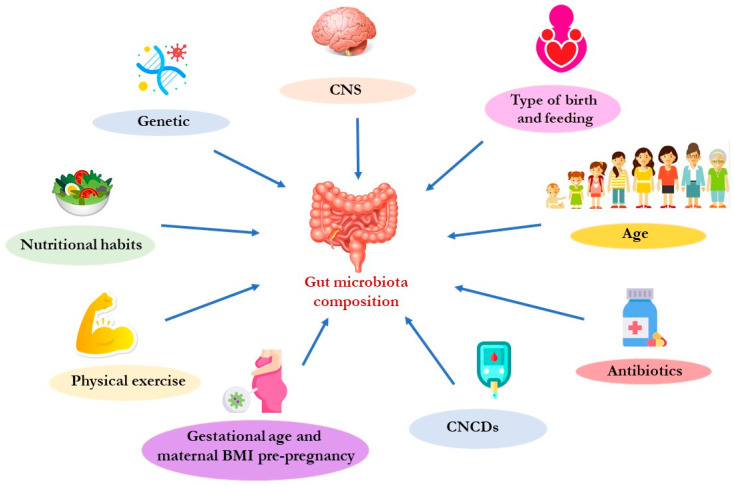
Main factors that can influence the gut microbiota composition. Abbreviation: BMI, body mass index; CNCDs, chronic non-communicable diseases; CNS, central nervous system.

The microbiota is an extremely heterogeneous and complex system, strongly modulated by external factors (Figure 2).

The composition of the gut microbiota changes in relation to age. In fact, recent studies reported that the microbiota begins to colonize the intestinal tract during intrauterine life [45,46]. In this regard, it has been demonstrated that placental microbiota can influence, during pregnancy, correct fetal growth and development [47]. The maintenance of a correct placental eubiosis is influenced by the nutritional habits of the mother during pregnancy. In particular, a diet rich in fats may induce the placental dysbiosis that has been associated with a high risk of developing metabolic syndrome in adults [48]. Moreover, placenta dysbiosis can damage the maternal hypothalamic–pituitary–adrenal (HPA) axis and can influence the circulating levels of 5-HT, inducing possible damage to fetal neuronal development [49]. In a child’s gastrointestinal (GI) tract, Bifidobacteria are the most abundant bacteria [50]. In adulthood, the bacterial composition appears to be greater both in terms of the number of microorganisms and the diversification of the taxa (called α-diversity). The gut microbiota α-diversity reflects the variability of species within the human intestine. Recent studies have highlighted how it is directly related to host health and how a poor α-diversity is associated with various CNCDs [51,52]. The gut microbiota changes that are observed in relation to age are mainly due to the switch from a liquid to a solid diet [53]. This switch causes the enrichment of bacterial flora, especially the families of *Lachnospiraceae* and *Ruminococcaceae* [43]. Throughout life, dietary habits influence the composition of the gut microbiota. Scientific studies have reported that bacteria belonging to the genus Clostridium (the main producers of butyrate, a short-chain fatty acid—SCFA) are more present in subjects following a Mediterranean diet (MD), while the subjects that follow a Western diet show fewer bacteria responsible for the degradation of fibers, such as Prevotella and Succinivibrio [54,55]. In the elderly, gut microbiota composition is less variable due to the reduction in food variety in the diet and the reduction in fiber intake, which results in a decrease in Firmicutes, (among these, the *Clostridium* cluster XIVa and *Feacalibacterium prausnitzii* are involved in saccharolytic fermentation), and in an increase in Proteobacteria [56]. This induces the reduced availability of SCFAs that, in turn, contributes to the aging and increased activity of proteolytic fermentation [57]. This phenomenon induces an enhanced production of gut-derived toxins, such as trimethylamine N-oxide (TMAO), p-cresyl sulfate (p-CS), indoxyl sulfate (IS), and indole-3 acetic acid (IAA). Moreover, an increase in proteolytic fermentation triggers low-grade chronic systemic inflammation, a factor related to the onset and progression of CNCDs and sarcopenia [27,58,59].

Other factors that influence the gut microbiota’s qualitative and quantitative compositions are (i) gestational age; (ii) antibiotic therapy, mostly in the perinatal period; (iii) mode of childbirth (vaginal or cesarean); (iv) type of feeding (breast feeding, artificial or mixed, composition and timing of complementary feeding); (v) pre-pregnancy body mass index (BMI); and maternal body weight increase during pregnancy [60,61].

(i) *Gestational age.* An interesting study analyzed the gut microbiota’s possible differences in full-term and in pre-term deliveries. Full-term infants are characterized by a greater abundance of *Bacteroides,* while pre-term infants show a greater abundance of *Lactobacillus* [62].
(ii) *Antibiotic therapy, especially in the perinatal period.* Antibiotics, especially broad-spectrum ones, qualitatively and quantitatively alter the gut microbiota’s composition [63]. Studies conducted on mice suggested complex mechanisms (endocrine and neurocrine) involved in the signaling between gut microbiota and the brain, which were induced by an excessive use of antibiotics, especially in children [64]. In fact, an excessive use of antibiotics promotes the gut colonization of *Clostridium difficile* [65], an opportunistic pathogen that can cause diarrhea, specifically in the case of the simultaneous intake of drugs that reduce the gut microbiota α-diversity [66,67].
(iii) *Mode of childbirth.* It has been shown that the microbiota of natural births is dominated by bacterial genera, such as *Bacteroides* (Bacteroidetes), bifidobacteria (Actinobatteria), lactobacilli (Firmicutes), and enterobacteria (Proteobacteria). On the contrary, the microbial pattern of Caesarean births is instead characterized by a qualitative–quantitative alteration in the gut microbiota’s composition [68]. Additionally, Caesarean births lack the Bacteroides species until 18 months of age, due to non-exposure to the maternal vaginal microbiota [69].
(iv) *Type of feeding.* The α-diversity appears to be lower in breastfed infants compared to formula-fed infants, and the gut microbiota composition shows differences. *Bifidobacterium* and Bacteroides are more abundant in breastfed infants, while Streptococcus and Enterococcus are more abundant in formula-fed infants [70].
(v) *Pre-pregnancy BMI and maternal body weight gain during pregnancy.* A recent study pointed out the differences in maternal gut microbiota composition based on pre-pregnancy weight and gestational weight gain. In fact, overweight/obese mothers, before pregnancy, are characterized by the presence of some taxa, such as the *Christensenellaceae* family and the genera *Lachnospira, Parabacteroides, Bifidobacterium*, and *Blautia.* Moreover, overweight or obese women before pregnancy show less α-diversity compared to non-overweight/non-obese women. This gut microbiota maternal pattern seems to not be related to the global differences in the infant gut microbiota within the first two years of life [71].

Recent studies demonstrated that physical activity is also able to modulate the gut microbiota, in terms of the composition and function [72]. On the contrary, physical inactivity has been repeatedly associated with alterations in the bacterial composition of the gut microbiota [73,74]. In particular, as described in the following paragraph, physical exercise is able to improve the body’s composition, positively modulating the gut microbiota pattern and stimulating SCFAs production [75]. Nevertheless, the benefits obtained during a period of continuous physical activity are lost if you return to a sedentary condition [76].

Furthermore, as previously described, CNCDs are, at the same time, both the cause and consequence of gut microbiota dysbiosis [32,77]. For example, in CKD patients, there is an alteration in gut permeability, which allows the passage of bacteria and bacterial material from the gut to the bloodstream. This phenomenon induces a chronic inflammatory state that exacerbates CKD itself. In CKD patients, an accumulation and increased production of gut-derived toxins can be observed (such as IS, TMAO, and p-CS, IAA). These toxins are associated with an increase in cardiovascular (CV) risk [27].

## 4. Migraine and Gut Dysbiosis

In recent decades, the literature demonstrates that the gut microbiota is involved in central nervous system (CNS) activities [78]. Over time, several studies have investigated and defined some of the mechanisms through which the brain is able to connect itself to the gut, defining the “gut–brain axis” [79].

The importance of this axis has been mainly highlighted in the studies that evaluated the causes underlying neurodegenerative and psychiatric diseases and neurodevelopment [80,81]. In particular, the CNS can influence the gut environment impacting on some intestinal functions, such as the regulation of gut movements, excretions, and the immune system [82]. The interaction between the intestine and brain is confirmed by a gut microbiota modification, which can impact on the CNS’s different functions [64,83].

The brain communicates with the intestine through numerous “roads” [64], such as (i) the immune system; (ii) SCFAs; (iii) the autonomic nervous system (vagal nerve); (iv) tryptophan metabolism, the precursor of the neurotransmitter serotonin; (v) neurotransmitters; (vi) gastric peptides produced by specialized endocrine cells, whose release is influenced by the microbiota itself; and (vii) the HPA axis [30,84].

Therefore, the intestine can be considered as the “second brain”, as it produces a series of neurotransmitters, such as serotonin and histamine [85]. Several studies demonstrated that low levels of cerebral serotonin were strongly associated with migraine. In particular, migraineurs have higher levels of cerebral serotonin during an acute pain attack compared with the periods between attacks [86,87]. In this regard, a symptomatic migraine-specific class of drugs commonly used in the clinical practice are triptans, serotonin-receptor agonists that can “recreate” an optimal brain neurotransmitter concentration by reducing the inflammation and, consequentially, the pain. Unfortunately, these medications are characterized by several side effects, such as cardiovascular dysfunction, GI disorders, muscle aches, drowsiness, tingling, and dermatological disorders [88].

Moreover, it was demonstrated that chronic headache patients presented higher histamine concentrations than healthy subjects, either during or between migraine attacks [89]. Previous studies have focused on the effects of antihistamines in the pain treatment behind the proven histamine’s role as a potent migraine trigger. However, this evidence is of a poor quality and often limited by frequent undesirable adverse events (i.e., excessive somnolence). Although there are positive reports about histamine H3-receptor agonists (H3Rs) that seem to effectively inhibit the histamine release in the CNS, they are not very credible because they are based on subtherapeutic analgesic dosages [90,91].

As previously described, nausea and vomiting are symptoms commonly associated with migraine. Moreover, patients with frequent migraine attacks often present GI symptoms, such as reflux, diarrhea, and constipation [92]. Starting from this observation, Camara-Lemarroy et al. highlighted the relationship between migraine and GI disorders [93]. This statement is strengthened by the fact that inflammatory bowel disease patients often suffer from migraine [94]. Gut dysbiosis seems to play a key role in the pathogenesis of migraine, since this intestinal alteration seems to induce an increased production of gut-derived inflammatory cytokines that can modulate the HPA axis [95]. Furthermore, these gut-derived inflammatory cytokines, in turn, appear to be involved in the modulation of vagus nerve activity, which, among other functions, innervates the intestinal free fatty acid-receptor 3. This receptor seems to be involved in the metabolic pathway of SCFAs [96]. Therefore, the mechanisms underlying the interaction between the gut microbiota and migraine are supported by several studies that highlight the direct and indirect evidence [97]. The former includes the fact that gut dysbiosis, associated with an impaired production of SCFAs, induces the release of pro-inflammatory cytokines, such as tumor necrosis factor (TNF)-α. The latter, in animal models, seems to regulate migraine-like pain [98]. The second ones are deduced from the scientific studies that suggest that nutritional approaches, use of probiotics, and stimulation of the vagus nerve can represent new therapeutic strategies for migraine, based on gut microbiota modulation [99,100,101]. Therefore, it is evident that there is a correlation between the gut microbiota and migraine. In fact, numerous studies both conducted on animal models and clinical trials highlighted a gut microbiota alteration in migraine patients compared to the healthy subjects. In this regard, it is important to understand whether the possible comorbidities (such as CNCDs) inducing dysbiosis can be a trigger for migraine and, at the same time, whether specific nutritional approaches or lifestyle changes can represent a valid and innovative adjuvant therapy for the treatment of migraine.

## 5. Migraine and Its Correlation with Chronic Non-Communicable Diseases

**Figure 3:**
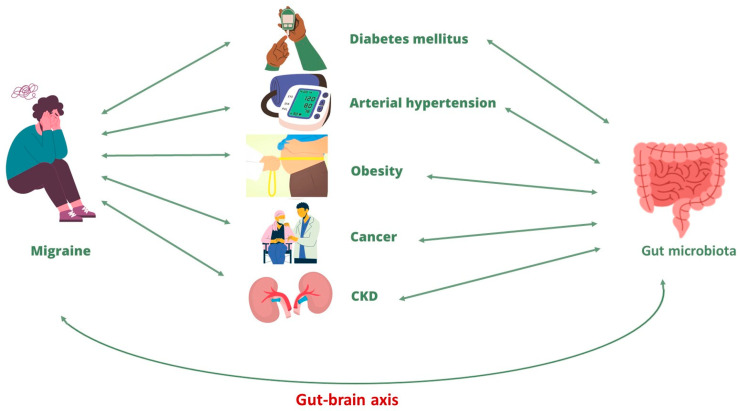
Correlation between migraine and chronic non-communicable diseases. Abbreviation: CKD, chronic kidney diseases.

The bi-directional correlation between migraine and the main CNCDs is already described in the literature, even if the mechanisms underlying this correlation are not fully clarified. A possible link between these pathological conditions could be represented by gut microbiota alteration, that is, by a condition known as dysbiosis. In fact, as widely described in the previous paragraphs, gut microbiota dysbiosis appears to represent both an important trigger for the onset of migraine and for its chronic development and, in turn, seems to be induced by the migraine itself. This relationship is possible thanks to the gut–brain axis, which allows the communication between the two organs. At the same time, an alteration in the gut microbiota has been broadly described in the literature in CNCDs patients. In fact, gut dysbiosis can be involved both in CNCDs progression and in chronic migraine onset in CNCDs patients (Figure 3). In this context, lifestyle changes and healthy nutritional approaches, capable of restoring gut eubiosis, can represent a valid adjuvant therapy to simultaneously improve the symptoms and the clinical evolution of the migraine and, consequently, the CNCDs’ clinical course.

### 5.1. Diabetes Mellitus and Migraine

The correlation between DM and migraine is currently controversial [102]. Some epidemiological studies suggest that migraine can be considered a non-traditional risk factor for DM onset and progression [103,104]. In fact, a study demonstrated that migraine attacks in DM patients may be related to the episodes of hypoglycemia that these patients often manifest. Moreover, it was supposed that a possible treatment able to counteract migraine episodes was optimal glycemic control with a contextual reduction in hypoglycemic episodes [102].

Moreover, at the same time, the insulin resistance that characterizes type-2 diabetes mellitus patients has been associated with migraine development. In particular, insulin resistance seems capable of significantly prolonging migraine attacks, but not their severity [105]. A possible explanation for this last correlation could be that insulin resistance, at the brain level, can lead to an impaired release of neurotransmitters. Moreover, insulin resistance and high blood glucose levels can cause a systemic inflammatory response that spreads to the peripheral and central nervous systems, inducing the neuroinflammation phenomenon [106,107]. In turn, neuroinflammation can damage the blood–brain barrier [108].

On the contrary, other studies showed an inverse correlation between these two pathological conditions, considering type-1 DM a protective factor against migraine. Although the exact mechanisms that underline this indirect relationship have not yet been sufficiently elucidated, it has been proposed that similarities in the genetic and biochemical pathways and in lifestyle could account for this [109,110,111]. To corroborate this observation, a recent epidemiological study conducted on over 70,000 French women highlighted a lower risk of developing type-2 DM in women with migraine compared to those without a migraine history [112].

### 5.2. Arterial Hypertension and Migraine

AH and headaches have been linked to each other in the medical literature [113]. In fact, it is well known how a hypertensive crisis, namely, a sudden increase in blood pressure, can occur with abrupt headaches [114]. Migraine and AH may share common mechanisms, such as endothelial dysfunction (ED), a deficiency of autonomic CV regulation, and renin–angiotensin–aldosterone system (RAAS) involvement [114]. Preventive anti-migraine effects were described for several antihypertensive drugs, such as beta-blockers, angiotensin-converting enzyme inhibitors, and angiotensin II-receptor blockers [115]. ED may be both one of the factors that increase the cerebrovascular and CV risks in migraine subjects and a determinant cause of AH development [116]. Nitric oxide (NO) plays a pivotal role in the pathogenesis of migraine; in fact, it is involved in the regulation of cerebral and extra-cerebral cranial blood flows and artery size. In migraineurs, the arteries are hypersensitive to NO, and it has been hypothesized that this phenomenon is one of the main triggers of migraine attacks [117].

Moreover, the RAAS can be another factor responsible for the clinical correlation between migraine and AH [118]. This might explain why some angiotensin-converting enzyme inhibitors and angiotensin II-receptor blockers are effective in preventing migraine, regardless of the presence of AH [119]. Notably, the presence of AH in a migraine patient increases the CV risk [120].

### 5.3. Obesity and Migraine

The WHO defines obesity as a condition characterized by an excessive presence of adipose tissue in the human body that induces a significant increase in CV mortality and morbidity in both sexes [121]. Obesity is commonly considered a consequence of excessive calorie intake, compared to energy expenditure, and the decrease in physical activity, very common habits in Western societies [122].

The clinical evidence shows that obesity can intensify the risk of episodic and chronic migraine and that the body weight reduction in obese subjects can decrease the intensity, frequency, and duration of migraine attacks [123].

The link between obesity and headaches is attributed to shared pathophysiological features [124]. For example, obese adult subjects showed increased calcitonin gene-related peptide (CGRP) plasma levels, which were also elevated in migraine patients [125]. Furthermore, an increase in proinflammatory cytokines, such as interleukin (IL)-6 and TNF-α, was reported in obese individuals and in the acute headache phase [126].

### 5.4. Cancer and Migraine

For most cancer types, there is no evidence to support a link between increased oncological risks in migraineurs [127], except for tumors of a neurological origin of which migraine can often represent the onset symptom. In contrast, a lower prevalence of GI cancers in migraine subjects has been described, compared to those without migraine [128].

Moreover, cancer patients may present an amplified risk of developing migraine. It is very important to recognize migraine causes in order to treat them promptly and exclude the presence of secondary headache causes (i.e., brain metastasis) [129]. Migraine induces a negative impact on the quality of life of cancer patients and, showing a very broad etiology, makes its diagnosis difficult [130]. For cancer patients, any change in pharmacological treatments, including chemotherapy, can trigger headaches [131].

### 5.5. CKD and Migraine

Several epidemiological studies have highlighted a direct correlation between CKD and migraine onset, even if the mechanisms underlying this correlation remain partially known. A study conducted by Wang et al. evaluated CKD incidence in a group of subjects with normal renal functions suffering from chronic migraine, compared to a group of subjects without migraine. The authors showed that CKD incidence was higher in subjects suffering from chronic migraine than in the control group. In particular, male gender, age, and nonsteroidal anti-inflammatory drugs (NSAIDs) abuse appeared to be independent risk factors for CKD onset [132]. This correlation seemed to be associated with ED secondary to a migraine, which would increase the risk of developing CKD [133]. Other factors that can lead to CKD onset in chronic migraine patients are blood pressure fluctuations and coexisting comorbidities [134]. A study evaluated the possible genetic link between the two pathological conditions; although, no overall genetic correlation was found, four specific genomic regions were identified that appeared to be related to the common pathogenic mechanisms underlying both migraine and CKDs. These mechanisms appeared to involve the cardiovascular system and endothelial function [133].

Hemodialysis-related migraine has been described in the literature. In fact, hemodialysis patients often suffer from headaches during or after hemodialysis (HD) treatment. The headache usually occurs in the first hour of an HD session and it resolves after a maximum of 72 h after HD treatment [135]. This phenomenon can be related to the electrolyte imbalance and blood pressure fluctuations that occur during dialysis treatment [136].

## 6. Possible Nutritional Approaches and Lifestyle Changes to Counteract Migraine

Proper nutrition patterns and a healthy lifestyle are useful to prevent and alleviate headache symptoms. This issue has a very ancient origin [137]. In fact, Ippocrate was aware of the relationship between the consumption of some foods and the onset of migraine [138]. Indeed, there are many foods that can cause painful attacks because they contain substances that are able to alter intracranial blood circulation [139], inducing vasodilation–vasoconstriction imbalance and a consequent headache. In this context, a healthy diet may represent a valid preventive therapeutic strategy for migraine [140].

### 6.1. Mediterranean Diet

The MD can be defined as a model of dietary habits adopted by the populations living in the Mediterranean area [141]. It is characterized by a high consumption of fresh fruit and vegetables, legumes, and complex carbohydrates, accompanied by a moderate consumption of seafoods and extra virgin olive oil (EVOO), as the main source of fats, and by a moderate consumption of wine [141]. There are numerous clinical studies that highlight how MD is able to reduce the risk of CNCDs onset and slow down their progression [142]. MD may also be considered an adjuvant approach to fight migraine in this patient population [143].

A meta-analysis highlighted the potential role of the MD in the fight against major neurodegenerative diseases [144]. This diet is rich in polyunsaturated fatty acids (PUFAs) and monounsaturated fatty acids (MUFAs) that seem to prevent the onset of Alzheimer’s and Parkinson’s diseases [145]. Moreover, the intake of PUFAs and specifically of eicosapentaenoic acid (EPA) seem to have antidepressant effects [146]. A recent meta-analysis analyzed 26 randomized double-blind placebo-controlled trials, highlighting the beneficial effects on the depression symptoms of EPA intake, at a dosage of ≤1 g/day, compared to a placebo [147]. Furthermore, as PUFAs are the main lipid-forming cerebral cortex, they are able to play an important role in higher cognitive processes and in learning [148].

Moreover, the MD is rich in polyphenols, vitamins C, E, B12, and B9, and carotenoids, and it is therefore able to counteract oxidative stress (OS) and lipid peroxidation, exerting cardioprotective and neuroprotective effects [149].

The MD seems to reduce chronic migraine symptoms [143]. In fact, a recent clinical study conducted on subjects aged between 18–64 years, who suffered from chronic migraines, highlighted how those who had a poor adherence to the MD developed more severe and frequent migraine attacks, compared to those who had a high adherence to the MD [150]. This symptom reduction appeared to be associated with the systemic inflammation decrease, mediated by MD typical foods. A Western diet, on the other hand, rich in pro-inflammatory foods, seems to be associated with an increase in migraine symptoms [151,152]. These associations seem to be related to the gut microbiota modification induced by the different nutritional patterns. In fact, it is known that the Western diet, characterized by a high dietary intake of salt, increases the *Firmicutes/Bacteroidetes* ratio, providing the conditions for gut dysbiosis. The latter induces a reduction in SCFA-producing bacteria, losing their important beneficial functions for the host [55]. On the other hand, the MD seems capable of positively modulating the gut microbiota by increasing its α-diversity [153].

The Western diet, rich in saturated fatty acids, is characterized by an increase in proteolytic fermentation, which leads to the production of gut-derived toxins, as previously described. Instead, the MD is characterized by an enhancement of saccharolytic fermentation that stimulates SCFAs production [27].

### 6.2. The Ketogenic Diet

The ketogenic diet (KD) was initially used as an adjuvant treatment for drug-resistant epilepsy and, to date, it is also widely used for rapid weight loss [154]. Its effects on neurodegenerative diseases, cognitive functions, and autistic spectrum disorders have also been studied. This nutritional approach is characterized by a high lipid intake, the right amounts of proteins, and a low energy intake from carbohydrates. The objective of KD is to reduce glycolysis and to stimulate the formation of endogenous ketones through the oxidation of fatty acids [155].

The increased concentration of ketones leads to a state of ketosis, so that these molecules take the place of glucose and can be used as the primary energy source by the brain. The KD can protect the brain from OS and can normalize the neuronal bioenergetics through the stimulation of mitochondrial biogenesis and the stabilization of synaptic functions [156].

The first piece of evidence that the KD was able to alleviate the symptoms of migraine dates back to the beginning of the last century. Since then, numerous clinical studies have been conducted on patients suffering from chronic headaches. These studies have shown that, through different mechanisms, the KD is able to reduce systemic inflammation [157] and OS [158] at the level of the CNS, and to positively modulate the gut microbiota [159], reducing the symptoms of migraine [160,161,162].

### 6.3. Probiotic and Prebiotic Supplementations

The WHO has defined probiotics as “living micro-organisms” capable of creating benefits to human health when administered in adequate quantities [163]. The main prebiotics belong to genera Lactobacilli and Bifidobacteria and their effects depend on the species and the strain, for example, *Lactobacillus rhamnosus* seems to be very effective in the treatment of GI disorders, such as infectious diarrhea in children, or in the prevention of antibiotic-induced diarrhea [164,165].

Probiotic integration can modulate chronic migraine symptoms [32,166]. The possible mechanisms of action are unclear and may include the stimulation of SCFAs production, the improvement of gut epithelial integrity, and the decrease in inflammation by the suppression of the kappa-B nuclear factor (NF-κB) pathway, lowering the levels of proinflammatory cytokines [167]. Probiotics can also increase the rate of gastric emptying and attenuate gastric stasis, a GI disorder commonly present in patients with migraine [168]. Several studies have shown that probiotics assumption can rebalance the gut microbiota’s composition, improve gut permeability, and prevent the onset of neurological disorders, such as migraine [169].

In some subjects, even some food allergies and intolerances can trigger migraine attacks. Conversely, a decrease in gut permeability can provide relief from migraine. Therefore, probiotics, thanks to an improvement of gut barrier functions, can also have beneficial effects on headache patients. New clinical studies are necessary to confirm this hypothesis [124,170].

Prebiotics are described as undigested substances capable of selectively stimulating the growth and/or the activity of one or a limited number of symbiotic intestinal bacteria. Prebiotics supplementation is able to restore gut eubiosis and can promote a reduction in migraine attacks [171]. Many studies focus on the effects induced by the two main prebiotics, galactogosharides (GOSs) and fructooligosaccharides (FOSs), demonstrating how these organic substances are able to reduce neuroinflammation and brain OS [172,173]. GOSs and FOSs seem to stimulate brain functions and synaptic plasticity towards the brain neurotrophic factor and receptors for N-methyl-D-aspartic acid (NMDA) [174].

### 6.4. Physical Activity

Physical activity, at the doses recommended by the WHO, seems to improve the quality of life. In particular, the WHO recommends a daily moderate-intensity aerobic physical activity of at least 150–300 min, or 75–150 min of vigorous-intensity aerobic physical activity for adults [175]. This physical activity seems to improve muscle mass, cardiorespiratory fitness, and bone health, and it appears to reduce the risks of AH, CV diseases, DM, various types of cancer (including breast and colon cancers), and depression [176]. Regarding this latter aspect, the mechanisms whereby physical exercise exerts its beneficial neurological effects are numerous, which include the regulation of the HPA axis, the promotion of an anti-inflammatory state, and the increase in neuroplasticity [177]. It is interesting to note that physical exercise can determine changes in the gut microbiota’s composition, restoring homeostasis and regulating energy expenditure [178]. Low-intensity physical exercise can affect GI tract functions, reducing the intestinal transit time and, thus, the contact time between pathogens and the GI mucus layer [179].

**Figure 4:**
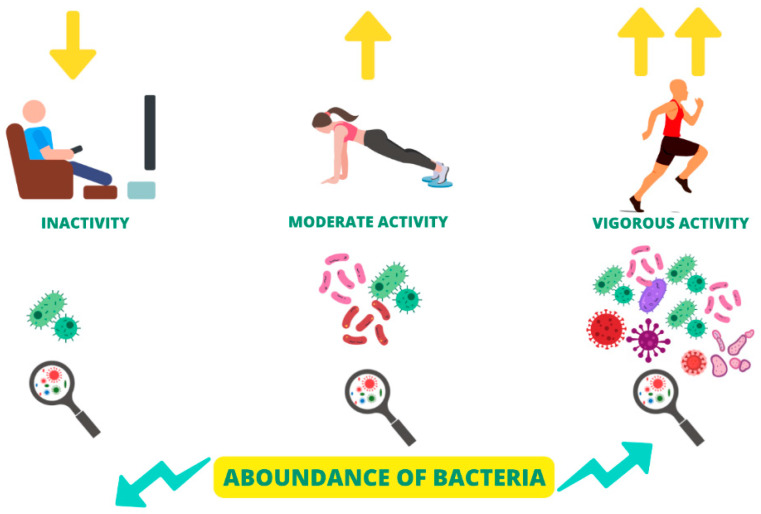
Modulation of gut microbiota composition and α-diversity by the physical activity degree.

Adapted physical activity can represent a valid non-pharmacological strategy for the clinical management of patients affected by CNCDs [180]. In fact, these patients, as previously illustrated, present an alteration in the gut microbiota, which induces and amplifies the chronic inflammatory state, which is, in turn, responsible for CNCDs progression [181]. Numerous studies suggest that adapted physical activity in CNCDs patients is able to both qualitatively and quantitatively modulate the gut microbiota’s composition, exerting important benefits on the patient’s quality of life and reducing CV risk [74]. Physical activity increases the abundance of gut microbiota bacteria and improves its quality (Figure 4) [182]. Studies on animal models suggest that physical activity is able to increase the abundance of the genus *Bacteroidetes* and decrease the genus *Firmicutes*, with a consequent reduction in the *Firmicutes*/*Bacteroidetes* ratio, improving the quality of the gut microbiota composition [74].

Thanks to the positive modulation of the gut microbiota by physical exercise, the latter, especially the aerobic type, seems able to prevent the onset of chronic migraine, rather than being able to reduce the symptoms of chronic headaches already existing. Several clinical studies, in fact, have highlighted how sedentary subjects are more likely to develop chronic migraine compared to active subjects [183]. On the other hand, other clinical studies showed that, in subjects who already suffered from migraine, intense physical exercise led to the exacerbation of this disorder [184].

### 6.5. Vitamin D Supplementation

It is well known that a vitamin D deficiency is associated with chronic pain, depression, and some neurological disorders [185]. The brain is characterized by an abundance of receptors for vitamin D and there is evidence of the non-skeletal role of vitamin D in the mechanisms that regulate inflammation, immunity, and neurotransmitter metabolism [186]. Vitamin D blood levels are related to sun exposure (depending on the latitude and outdoor activities), dietary intake, and genetic components [187]. Migraine patients tend to avoid sunlight because of photophobia during migraine attacks. Moreover, it is known that reduced physical activity and a sedentary lifestyle, conducted indoors, greatly increase the risk of vitamin D deficiency [188].

Several studies have reported that vitamin D low-serum levels may be associated with an increased risk of migraines/headaches [189]. In addition, it has been suggested that the prevalence of a deficiency/insufficiency of vitamin D may be greater in patients suffering from migraines/headaches when compared with subjects without headaches [190].

### 6.6. Other Vitamin Supplementations

In patients with very intense migraine, a lack of vitamin B12 is often observed [191]. Vitamin B12 deficiency in association with hyperhomocysteinemia causes damage to endothelial cells, increasing free radical levels, which may be related to migraine episode generation [192]. Recent studies suggested that a vitamin B12 deficiency in chronic migraine can be induced by the frequent use of analgesics, which may alter vitamin B12 absorption [193].

Furthermore, studies on riboflavin have shown that the intake of this vitamin is effective in the prophylaxis of migraine, thus reducing the frequency of attacks [194].

Emerging evidence shows the statistically significant role of thiamine (vitamin B1) in migraine mitigation [195,196]. Thiamine has been shown to be particularly important in the regulation of brain levels of serotonin; abnormalities in the function of serotonin seem to be directly involved in the pathophysiology of migraine [86].

Vitamin K2 supplementation can also play a potential role in patients with migraine [197].

### 6.7. Iron Supplementation

Dietary iron is the primary source of iron in the body [198]. Dietary iron intake has different effects on migraine in women of different ages, and these different effects may be due to age-related menstrual changes [199]. Higher serum levels of ferritin in women over 50 years of age may have a protective effect against migraine [200]. The recommended dietary allowance (RDA) for iron is 18 mg/day for females; however, as evidenced by numerous scientific studies, the average dietary intake of iron for women aged 20 to 50 years old is lower than the RDA [201].

To verify the frequency of migraine attacks in patients with iron deficiency anemia, a study was conducted on 127 subjects who underwent validated tests on migraine, anxiety, depression, and quality of life. The results obtained showed that almost 80% of patients suffered from recurring headaches and that they were often smokers and showed low hemoglobin levels and low mean corpuscular volume values. Moreover, most patients with an iron deficiency presented depression, anxiety, and a poor quality of life [202].

### 6.8. Polyphenol-Rich Foods Consumption

Polyphenols are important constituents of plant-based foods, closely related to the main sensory and beneficial properties of fruit, vegetables, and their derivatives. Polyphenols can be classified into four main categories (flavonoids, phenolic acids, stilbenes, and lignans) based on the number of phenolic rings and other structural elements that bind these rings together [203]. A diet rich in polyphenols, especially flavanones and lignans, has been associated in the literature with reduced migraine severity. A lower intake of phenols and flavonoids, on the other hand, seems to correlate with more severe migraine attacks. Encouraging the consumption of polyphenol-rich foods, such as fruit, vegetables, and EVOO, can represent a valid adjuvant strategy to counteract migraine [204]. The beneficial analgesic action of polyphenols seems to be mainly exerted by their antioxidant properties. Indeed, the literature studies suggest that OS may play a significant role in the pathogenesis of migraine. The antioxidant compounds contained in foods seem to be able to prevent OS by inhibiting the initiation and propagation of the oxidative chain reaction, which is at the basis of the migraine attack [205].

One of the most controversial polyphenol-rich foods related to migraine onset is chocolate (rich in catechins, flavonols, anthocyanins, and procyanidins). Numerous studies have evaluated the possibility that the consumption of chocolate can stimulate migraine attack onset in subjects who are predisposed to it; however, the pathophysiological mechanisms underlying this correlation have not been elucidated and there is currently insufficient evidence to establish if chocolate is a real migraine trigger [206].

### 6.9. Magnesium Supplementation

Magnesium is an essential mineral that plays an important role in nerve function [207]. Recent studies have shown, for the general population, a lower average magnesium consumption level than the dietary recommendations, suggesting that a low intake of magnesium may be associated with migraine and that a good percentage of migraine subjects may suffer from a magnesium deficiency [208]. Magnesium inhibits neuronal overexcitation and vasospasm, reduces the formation of inflammatory substances, and improves mitochondrial oxidative phosphorylation and serotonin receptor transmission [209]. At present, most studies on magnesium as a possible preventive treatment for migraine are limited to oral food supplements, not considering magnesium-rich foods [208]. However, most of these oral food supplements have several limitations and side effects, such as GI disorders, nephrolithiasis, and an increased risk of CV disease [210].

### 6.10. Abstention from Histamine-Rich Food Consumption

Histamine (2-[3H-imidazol-4-yl]ethanamine) activation causes a number of vascular phenomena, which can result in a migraine attack [90,211]. Histamine is a substance that is regularly produced by our body within immune cells [212]. It is a chemical mediator within our body that performs two important functions: a mediator of inflammatory and allergic reactions and a neurotransmitter [212]. Several host factors, in addition to genetic factors, may influence histamine/receptor effects, including the gut microbiota composition, gender, aging, autoimmune diseases, cancer, and pulmonary diseases [213].

Histamine is also a biogenic amine that can be found in many foods; in particular, it may be present in high levels in those foods, often referred to as triggers of migraine in susceptible individuals [214]. The relationship between the diet and migraine has long been controversial and it based on the association between the consumption of certain foods and the manifestation of migraine pain [215]. Many of the foods in question are potentially rich in biologically active amines: histamine, tyramine, and others [216]. Because of the genetic origin, some subjects produce low levels of diaminossidase (DAO) and this means that an excess of histamine is not neutralized and it causes, among other issues, migraine [217]. Mutations involving genes responsible for producing DAO (AOC1 on chromosome 7) can increase the susceptibility to histamine-intolerance development [218]. Some drugs, including NSAIDS, antidepressants, immunomodulators, antiarrhythmics, and other substances (e.g., acetylcysteine, clavulan acid, metoclopramide, and verapamil), may decrease the threshold of tolerance to histamines [219]. Alcohol consumption, in particular, red wine, is a powerful inhibitor of DAO because it also contains, in addition to high levels of histamine, other classes of biogenic amines, such as tyramine and sulfites, which compete with the histamine for binding to the active site of the enzyme [220].

## 7. Conclusions

Migraine development seems to be partially related to the condition of gut dysbiosis. In fact, it can lead to a reduction in SCFAs production and to a concomitant increase in gut-derived inflammatory cytokines, which can influence CNS activities and, in turn, cause migraine. Moreover, there seems to be a bi-directional correlation between migraine and the risk of CNCDs onset and progression, where the gut microbiota plays a pivotal role.

Besides, numerous studies highlight an association between specific nutritional patterns and lifestyles related to gut eubiosis restoration and the prevention of migraine attacks. Therefore, healthy nutritional habits (such as a MD), an appropriate choice of foods both in quantitative and qualitative terms, oral food supplements administration (such as prebiotics and probiotics), and a constant physical exercise seem to be effective adjuvant strategies for migraine prevention.

The main limitations of this narrative review are certainly to be found in the lacking of this scientific field. In fact, the literature and clinical trials, regarding the link between CNCDs and migraine and the possible role played by gut dysbiosis, are limited and, above all, controversial. To better clarify these possible correlations, it would be useful to perform randomized clinical trials on CNCDs patients with migraine, evaluating the potential presence of gut dysbiosis and monitoring how it can influence the progression of these pathological conditions. Moreover, it would be interesting to examine the positive impacts of possible therapeutic approaches, based on a personalized diet and oral food supplements, formulated *ad hoc.*

## Data Availability

Not applicable.

## Abbreviations

5-HT	5-hydroxytryptamine
AH	Arterial Hypertension
BDNF	Brain Neurotrophic Factor
BMI	Body Mass Index
CGRP	Calcitonin Gene-Related Peptide
CKD	Chronic Kidney Disease
CNCDs	Chronic Non-Communicable Diseases
CNS	Central Nervous System
CV	Cardiovascular
DAO	Diaminossidase
DM	Dibetes Mellitus
ED	Endothelial Dysfunction
EPA	Aicosapentaenoic Acid
EVOO	Extra Virgin Olive Oil
FOSs	Fructooligosaccharides
GI	Gastrointestinal
GOSs	Galactogosharides
HPA	Hypothalamic–Pituitary–Adrenal
H3R	Histamine H3 Receptor Agonist
IAA	Indole 3 Acetic Acid
IL	Interleukin
IS	Indoxyl Sulphate
KD	Ketogenic Diet
MD	Mediteranean Diet
MUFAs	Monounsaturated Fatty Acids
NF-κB	Kappa-B Nuclear Factor
NMDA	N-Methyl-D-Aspartic Acid
NO	Nitric Oxide
NSAIDs	Nonsteroidal Anti-Inflammatory Drugs
OS	Oxidative Stress
p-CS	P-Cresyl Sulfate
PUFAs	Polyunsaturated Fatty Acids
RAAS	Renin–Angiotensin–Aldosterone System
RDA	Recommended Dietary Allowance
SCFAs	Short Chain Fatty Acids
TMAO	Trimethylamine N-Oxide
TNF	Tumor Necrosis Factor
WHO	World Health Organization

## References

[1] Burstein R., Noseda R., Borsook D. (2015). Migraine: Multiple processes, complex pathophysiology. J. Neurosci..

[2] Stovner L.J., Hagen K., Linde M., Steiner T.J. (2022). The global prevalence of headache: An update, with analysis of the influences of methodological factors on prevalence estimates. J. Headache Pain.

[3] Steiner T.J., Stovner L.J., Vos T., Jensen R., Katsarava Z. (2018). Migraine is first cause of disability in under 50s: Will health politicians now take notice?. J. Headache Pain.

[4] Cieza A., Causey K., Kamenov K., Hanson S.W., Chatterji S., Vos T. (2021). Global estimates of the need for rehabilitation based on the Global Burden of Disease study 2019: A systematic analysis for the Global Burden of Disease Study 2019. Lancet.

[5] Rossi M.F., Tumminello A., Marconi M., Gualano M.R., Santoro P.E., Malorni W., Moscato U. (2022). Sex and gender differences in migraines: A narrative review. Neurol. Sci..

[6] Sacco S., Lampl C., van den Brink A.M., Caponnetto V., Braschinsky M., Ducros A., Little P., Pozo-Rosich P., Reuter U., de la Torre E.R. (2021). Burden and attitude to resistant and refractory migraine: A survey from the European Headache Federation with the endorsement of the European Migraine & Headache Alliance. J. Headache Pain.

[7] Tonini M.C., Fiorencis A., Iannacchero R., Zampolini M., Cappuccio A., Raddino R., Grillo E., Albanese M., Allais G., Bassano M.A. (2021). Narrative Medicine to integrate patients’, caregivers’ and clinicians’ migraine experiences: The DRONE multicentre project. Neurol. Sci..

[8] Burch R.C., Buse D.C., Lipton R.B. (2019). Migraine: Epidemiology, Burden, and Comorbidity. Neurol. Clin..

[9] Paredes S., Cantillo S., Candido K.D., Knezevic N.N. (2019). An Association of Serotonin with Pain Disorders and Its Modulation by Estrogens. Int. J. Mol. Sci..

[10] Calhoun A.H., Batur P. (2017). Combined hormonal contraceptives and migraine: An update on the evidence. Cleve. Clin. J. Med..

[11] Calhoun A.H. (2017). Hormonal Contraceptives and Migraine With Aura-Is There Still a Risk?. Headache.

[12] Lee A., Syed Y.Y. (2022). Estetrol/Drospirenone: A Review in Oral Contraception. Drugs.

[13] Gasparini C.F., Smith R.A., Griffiths L.R. (2017). Genetic and biochemical changes of the serotonergic system in migraine pathobiology. J. Headache Pain.

[14] Zhang Q., Shao A., Jiang Z., Tsai H., Liu W. (2019). The exploration of mechanisms of comorbidity between migraine and depression. J. Cell Mol. Med..

[15] Peres M.F.P., Mercante J.P.P., Tobo P.R., Kamei H., Bigal M.E. (2017). Anxiety and depression symptoms and migraine: A symptom-based approach research. J. Headache Pain.

[16] Stubberud A., Buse D.C., Kristoffersen E.S., Linde M., Tronvik E. (2021). Is there a causal relationship between stress and migraine? Current evidence and implications for management. J. Headache Pain.

[17] Radat F. (2013). Stress and migraine. Rev. Neurol..

[18] Klan T., Diezemann-Prossdorf A., Guth A.L., Gaul C., Witthoft M. (2023). Fear of migraine attacks: Diagnosis and treatment. Schmerz.

[19] Tepper S.J. (2012). Medication-overuse headache. Continuum.

[20] Tfelt-Hansen P.C., Tfelt-Hansen J. (2009). Nitroglycerin headache and nitroglycerin-induced primary headaches from 1846 and onwards: A historical overview and an update. Headache.

[21] Lance J.W. (1991). 5-Hydroxytryptamine and its role in migraine. Eur. Neurol..

[22] Connor J.P., Stjepanovic D., Budney A.J., Le Foll B., Hall W.D. (2022). Clinical management of cannabis withdrawal. Addiction.

[23] Farooque U., Okorie N., Kataria S., Shah S.F., Bollampally V.C. (2020). Cocaine-Induced Headache: A Review of Pathogenesis, Presentation, Diagnosis, and Management. Cureus.

[24] Takahashi T.T., Ornello R., Quatrosi G., Torrente A., Albanese M., Vigneri S., Guglielmetti M., Maria De Marco C., Dutordoir C., Colangeli E. (2021). Medication overuse and drug addiction: A narrative review from addiction perspective. J. Headache Pain.

[25] Marmura M.J. (2018). Triggers, Protectors, and Predictors in Episodic Migraine. Curr. Pain Headache Rep..

[26] Minen M.T., Begasse De Dhaem O., Kroon Van Diest A., Powers S., Schwedt T.J., Lipton R., Silbersweig D. (2016). Migraine and its psychiatric comorbidities. J. Neurol. Neurosurg. Psychiatry.

[27] Noce A., Marchetti M., Marrone G., Di Renzo L., Di Lauro M., Di Daniele F., Albanese M., Di Daniele N., De Lorenzo A. (2022). Link between gut microbiota dysbiosis and chronic kidney disease. Eur. Rev. Med. Pharmacol. Sci..

[28] Noce A., Marrone G., Di Daniele F., Ottaviani E., Wilson Jones G., Bernini R., Romani A., Rovella V. (2019). Impact of Gut Microbiota Composition on Onset and Progression of Chronic Non-Communicable Diseases. Nutrients.

[29] Ceppa F.A., Izzo L., Sardelli L., Raimondi I., Tunesi M., Albani D., Giordano C. (2020). Human Gut-Microbiota Interaction in Neurodegenerative Disorders and Current Engineered Tools for Its Modeling. Front. Cell Infect. Microbiol..

[30] Cryan J.F., O’Riordan K.J., Cowan C.S.M., Sandhu K.V., Bastiaanssen T.F.S., Boehme M., Codagnone M.G., Cussotto S., Fulling C., Golubeva A.V. (2019). The Microbiota-Gut-Brain Axis. Physiol. Rev..

[31] Matijasic M., Mestrovic T., Paljetak H.C., Peric M., Baresic A., Verbanac D. (2020). Gut Microbiota beyond Bacteria-Mycobiome, Virome, Archaeome, and Eukaryotic Parasites in IBD. Int. J. Mol. Sci..

[32] Hills R.D., Pontefract B.A., Mishcon H.R., Black C.A., Sutton S.C., Theberge C.R. (2019). Gut Microbiome: Profound Implications for Diet and Disease. Nutrients.

[33] Gonzalez-Sarrias A., Romo-Vaquero M., Garcia-Villalba R., Cortes-Martin A., Selma M.V., Espin J.C. (2018). The Endotoxemia Marker Lipopolysaccharide-Binding Protein is Reduced in Overweight-Obese Subjects Consuming Pomegranate Extract by Modulating the Gut Microbiota: A Randomized Clinical Trial. Mol. Nutr. Food Res..

[34] Bjorksten B. (2006). The gut microbiota: A complex ecosystem. Clin. Exp. Allergy.

[35] Chakaroun R.M., Massier L., Kovacs P. (2020). Gut Microbiome, Intestinal Permeability, and Tissue Bacteria in Metabolic Disease: Perpetrators or Bystanders?. Nutrients.

[36] Rodriguez J.M., Murphy K., Stanton C., Ross R.P., Kober O.I., Juge N., Avershina E., Rudi K., Narbad A., Jenmalm M.C. (2015). The composition of the gut microbiota throughout life, with an emphasis on early life. Microb. Ecol. Health Dis..

[37] Thursby E., Juge N. (2017). Introduction to the human gut microbiota. Biochem. J..

[38] Mackowiak P.A. (2013). Recycling metchnikoff: Probiotics, the intestinal microbiome and the quest for long life. Front. Public Health.

[39] Belkaid Y., Hand T.W. (2014). Role of the microbiota in immunity and inflammation. Cell.

[40] Rowland I., Gibson G., Heinken A., Scott K., Swann J., Thiele I., Tuohy K. (2018). Gut microbiota functions: Metabolism of nutrients and other food components. Eur. J. Nutr..

[41] Miller I. (2018). The gut-brain axis: Historical reflections. Microb. Ecol. Health Dis..

[42] Wang W.L., Xu S.Y., Ren Z.G., Tao L., Jiang J.W., Zheng S.S. (2015). Application of metagenomics in the human gut microbiome. World J. Gastroenterol..

[43] Rinninella E., Raoul P., Cintoni M., Franceschi F., Miggiano G.A.D., Gasbarrini A., Mele M.C. (2019). What is the Healthy Gut Microbiota Composition? A Changing Ecosystem across Age, Environment, Diet, and Diseases. Microorganisms.

[44] Bull M.J., Plummer N.T. (2014). Part 1: The Human Gut Microbiome in Health and Disease. Integr. Med..

[45] Walker R.W., Clemente J.C., Peter I., Loos R.J.F. (2017). The prenatal gut microbiome: Are we colonized with bacteria in utero?. Pediatr. Obes..

[46] Tanaka M., Nakayama J. (2017). Development of the gut microbiota in infancy and its impact on health in later life. Allergol. Int..

[47] Nyangahu D.D., Jaspan H.B. (2019). Influence of maternal microbiota during pregnancy on infant immunity. Clin. Exp. Immunol..

[48] Easton Z.J.W., Regnault T.R.H. (2020). The Impact of Maternal Body Composition and Dietary Fat Consumption upon Placental Lipid Processing and Offspring Metabolic Health. Nutrients.

[49] Kapoor A., Dunn E., Kostaki A., Andrews M.H., Matthews S.G. (2006). Fetal programming of hypothalamo-pituitary-adrenal function: Prenatal stress and glucocorticoids. J. Physiol..

[50] Arboleya S., Watkins C., Stanton C., Ross R.P. (2016). Gut Bifidobacteria Populations in Human Health and Aging. Front. Microbiol..

[51] Manor O., Dai C.L., Kornilov S.A., Smith B., Price N.D., Lovejoy J.C., Gibbons S.M., Magis A.T. (2020). Health and disease markers correlate with gut microbiome composition across thousands of people. Nat. Commun..

[52] Pickard J.M., Zeng M.Y., Caruso R., Nunez G. (2017). Gut microbiota: Role in pathogen colonization, immune responses, and inflammatory disease. Immunol. Rev..

[53] Hagerty S.L., Hutchison K.E., Lowry C.A., Bryan A.D. (2020). An empirically derived method for measuring human gut microbiome alpha diversity: Demonstrated utility in predicting health-related outcomes among a human clinical sample. PLoS ONE.

[54] Conlon M.A., Bird A.R. (2014). The impact of diet and lifestyle on gut microbiota and human health. Nutrients.

[55] Canale M.P., Noce A., Di Lauro M., Marrone G., Cantelmo M., Cardillo C., Federici M., Di Daniele N., Tesauro M. (2021). Gut Dysbiosis and Western Diet in the Pathogenesis of Essential Arterial Hypertension: A Narrative Review. Nutrients.

[56] Kim S., Jazwinski S.M. (2018). The Gut Microbiota and Healthy Aging: A Mini-Review. Gerontology.

[57] Salazar N., Arboleya S., Fernandez-Navarro T., de Los Reyes-Gavilan C.G., Gonzalez S., Gueimonde M. (2019). Age-Associated Changes in Gut Microbiota and Dietary Components Related with the Immune System in Adulthood and Old Age: A Cross-Sectional Study. Nutrients.

[58] Lin X., Liang W., Li L., Xiong Q., He S., Zhao J., Guo X., Xiang S., Zhang P., Wang H. (2022). The Accumulation of Gut Microbiome-derived Indoxyl Sulfate and P-Cresyl Sulfate in Patients With End-stage Renal Disease. J. Ren. Nutr..

[59] Noce A., Marrone G., Ottaviani E., Guerriero C., Di Daniele F., Pietroboni Zaitseva A., Di Daniele N. (2021). Uremic Sarcopenia and Its Possible Nutritional Approach. Nutrients.

[60] Raspini B., Vacca M., Porri D., De Giuseppe R., Calabrese F.M., Chieppa M., Liso M., Cerbo R.M., Civardi E., Garofoli F. (2021). Early Life Microbiota Colonization at Six Months of Age: A Transitional Time Point. Front. Cell. Infect. Microbiol..

[61] Zhou L., Xiao X. (2018). The role of gut microbiota in the effects of maternal obesity during pregnancy on offspring metabolism. Biosci. Rep..

[62] Fouhy F., Watkins C., Hill C.J., O’Shea C.A., Nagle B., Dempsey E.M., O’Toole P.W., Ross R.P., Ryan C.A., Stanton C. (2019). Perinatal factors affect the gut microbiota up to four years after birth. Nat. Commun..

[63] Ramirez J., Guarner F., Bustos Fernandez L., Maruy A., Sdepanian V.L., Cohen H. (2020). Antibiotics as Major Disruptors of Gut Microbiota. Front. Cell. Infect. Microbiol..

[64] Carabotti M., Scirocco A., Maselli M.A., Severi C. (2015). The gut-brain axis: Interactions between enteric microbiota, central and enteric nervous systems. Ann. Gastroenterol..

[65] Perez-Cobas A.E., Moya A., Gosalbes M.J., Latorre A. (2015). Colonization Resistance of the Gut Microbiota against Clostridium difficile. Antibiotics.

[66] Langdon A., Crook N., Dantas G. (2016). The effects of antibiotics on the microbiome throughout development and alternative approaches for therapeutic modulation. Genome Med..

[67] Elvers K.T., Wilson V.J., Hammond A., Duncan L., Huntley A.L., Hay A.D., van der Werf E.T. (2020). Antibiotic-induced changes in the human gut microbiota for the most commonly prescribed antibiotics in primary care in the UK: A systematic review. BMJ Open.

[68] Coelho G.D.P., Ayres L.F.A., Barreto D.S., Henriques B.D., Prado M., Passos C.M.D. (2021). Acquisition of microbiota according to the type of birth: An integrative review. Rev. Lat. Am. Enfermagem..

[69] Mitchell C.M., Mazzoni C., Hogstrom L., Bryant A., Bergerat A., Cher A., Pochan S., Herman P., Carrigan M., Sharp K. (2020). Delivery Mode Affects Stability of Early Infant Gut Microbiota. Cell Rep. Med..

[70] Ma J., Li Z., Zhang W., Zhang C., Zhang Y., Mei H., Zhuo N., Wang H., Wang L., Wu D. (2020). Comparison of gut microbiota in exclusively breast-fed and formula-fed babies: A study of 91 term infants. Sci. Rep..

[71] Stanislawski M.A., Dabelea D., Wagner B.D., Sontag M.K., Lozupone C.A., Eggesbo M. (2017). Pre-pregnancy weight, gestational weight gain, and the gut microbiota of mothers and their infants. Microbiome.

[72] Dorelli B., Galle F., De Vito C., Duranti G., Iachini M., Zaccarin M., Preziosi Standoli J., Ceci R., Romano F., Liguori G. (2021). Can Physical Activity Influence Human Gut Microbiota Composition Independently of Diet? A Systematic Review. Nutrients.

[73] Dziewiecka H., Buttar H.S., Kasperska A., Ostapiuk-Karolczuk J., Domagalska M., Cichon J., Skarpanska-Stejnborn A. (2022). Physical activity induced alterations of gut microbiota in humans: A systematic review. BMC Sports Sci. Med. Rehabil..

[74] Noce A., Tranchita E., Marrone G., Grazioli E., Di Lauro M., Murri A., Vanni G., Della Morte Canosci D., Di Daniele N., Parisi A. (2023). The possible role of physical activity in the modulation of gut microbiota in chronic kidney disease and its impact on cardiovascular risk: A narrative review. Eur. Rev. Med. Pharmacol. Sci..

[75] Aragon-Vela J., Solis-Urra P., Ruiz-Ojeda F.J., Alvarez-Mercado A.I., Olivares-Arancibia J., Plaza-Diaz J. (2021). Impact of Exercise on Gut Microbiota in Obesity. Nutrients.

[76] Katzmarzyk P.T. (2010). Physical activity, sedentary behavior, and health: Paradigm paralysis or paradigm shift?. Diabetes.

[77] Vijay A., Valdes A.M. (2022). Role of the gut microbiome in chronic diseases: A narrative review. Eur. J. Clin. Nutr..

[78] Ojeda J., Avila A., Vidal P.M. (2021). Gut Microbiota Interaction with the Central Nervous System throughout Life. J. Clin. Med..

[79] Martin C.R., Osadchiy V., Kalani A., Mayer E.A. (2018). The Brain-Gut-Microbiome Axis. Cell. Mol. Gastroenterol. Hepatol..

[80] Novellino F., Sacca V., Donato A., Zaffino P., Spadea M.F., Vismara M., Arcidiacono B., Malara N., Presta I., Donato G. (2020). Innate Immunity: A Common Denominator between Neurodegenerative and Neuropsychiatric Diseases. Int. J. Mol. Sci..

[81] Maiuolo J., Gliozzi M., Musolino V., Carresi C., Scarano F., Nucera S., Scicchitano M., Oppedisano F., Bosco F., Ruga S. (2021). The Contribution of Gut Microbiota-Brain Axis in the Development of Brain Disorders. Front. Neurosci..

[82] Kappeter A., Sipos D., Varga A., Vigvari S., Halda-Kiss B., Peterfi Z. (2023). Migraine as a Disease Associated with Dysbiosis and Possible Therapy with Fecal Microbiota Transplantation. Microorganisms.

[83] Zhu X., Han Y., Du J., Liu R., Jin K., Yi W. (2017). Microbiota-gut-brain axis and the central nervous system. Oncotarget.

[84] Socala K., Doboszewska U., Szopa A., Serefko A., Wlodarczyk M., Zielinska A., Poleszak E., Fichna J., Wlaz P. (2021). The role of microbiota-gut-brain axis in neuropsychiatric and neurological disorders. Pharmacol. Res..

[85] Ochoa-Reparaz J., Kasper L.H. (2016). The Second Brain: Is the Gut Microbiota a Link between Obesity and Central Nervous System Disorders?. Curr. Obes. Rep..

[86] Hamel E. (2007). Serotonin and migraine: Biology and clinical implications. Cephalalgia.

[87] Deen M., Christensen C.E., Hougaard A., Hansen H.D., Knudsen G.M., Ashina M. (2017). Serotonergic mechanisms in the migraine brain—A systematic review. Cephalalgia.

[88] Ferrari M.D., Goadsby P.J., Roon K.I., Lipton R.B. (2002). Triptans (serotonin, 5-HT1B/1D agonists) in migraine: Detailed results and methods of a meta-analysis of 53 trials. Cephalalgia.

[89] Heatley R.V., Denburg J.A., Bayer N., Bienenstock J. (1982). Increased plasma histamine levels in migraine patients. Clin. Allergy.

[90] Worm J., Falkenberg K., Olesen J. (2019). Histamine and migraine revisited: Mechanisms and possible drug targets. J. Headache Pain.

[91] Yuan H., Silberstein S.D. (2018). Histamine and Migraine. Headache.

[92] Aamodt A.H., Stovner L.J., Hagen K., Zwart J.A. (2008). Comorbidity of headache and gastrointestinal complaints. The Head-HUNT Study. Cephalalgia.

[93] Camara-Lemarroy C.R., Rodriguez-Gutierrez R., Monreal-Robles R., Marfil-Rivera A. (2016). Gastrointestinal disorders associated with migraine: A comprehensive review. World J. Gastroenterol..

[94] Chang F.Y., Lu C.L. (2013). Irritable bowel syndrome and migraine: Bystanders or partners?. J. Neurogastroenterol. Motil..

[95] Zanos T.P., Silverman H.A., Levy T., Tsaava T., Battinelli E., Lorraine P.W., Ashe J.M., Chavan S.S., Tracey K.J., Bouton C.E. (2018). Identification of cytokine-specific sensory neural signals by decoding murine vagus nerve activity. Proc. Natl. Acad. Sci. USA.

[96] Cook T.M., Gavini C.K., Jesse J., Aubert G., Gornick E., Bonomo R., Gautron L., Layden B.T., Mansuy-Aubert V. (2021). Vagal neuron expression of the microbiota-derived metabolite receptor, free fatty acid receptor (FFAR3), is necessary for normal feeding behavior. Mol. Metab..

[97] Crawford J., Liu S., Tao F. (2022). Gut microbiota and migraine. Neurobiol. Pain.

[98] Tang Y., Liu S., Shu H., Yanagisawa L., Tao F. (2020). Gut Microbiota Dysbiosis Enhances Migraine-Like Pain Via TNFalpha Upregulation. Mol. Neurobiol..

[99] David L.A., Materna A.C., Friedman J., Campos-Baptista M.I., Blackburn M.C., Perrotta A., Erdman S.E., Alm E.J. (2014). Host lifestyle affects human microbiota on daily timescales. Genome Biol..

[100] Singh R.K., Chang H.W., Yan D., Lee K.M., Ucmak D., Wong K., Abrouk M., Farahnik B., Nakamura M., Zhu T.H. (2017). Influence of diet on the gut microbiome and implications for human health. J. Transl. Med..

[101] Ramsden C.E., Zamora D., Faurot K.R., MacIntosh B., Horowitz M., Keyes G.S., Yuan Z.X., Miller V., Lynch C., Honvoh G. (2021). Dietary alteration of n-3 and n-6 fatty acids for headache reduction in adults with migraine: Randomized controlled trial. BMJ.

[102] Haghighi F.S., Rahmanian M., Namiranian N., Arzaghi S.M., Dehghan F., Chavoshzade F., Sepehri F. (2015). Migraine and type 2 diabetes; is there any association?. J. Diabetes Metab. Disord..

[103] Lopez-de-Andres A., Luis Del Barrio J., Hernandez-Barrera V., de Miguel-Diez J., Jimenez-Trujillo I., Martinez-Huedo M.A., Jimenez-Garcia R. (2018). Migraine in adults with diabetes; is there an association? Results of a population-based study. Diabetes Metab. Syndr. Obes..

[104] Cheon D.Y., Han K., Yang Y.S., Kim Y., Lee S.H., Kim C., Sohn J.H., Oh M.S., Lee B.C., Lee M. (2022). Associations between migraine and major cardiovascular events in type 2 diabetes mellitus. Cardiovasc. Diabetol..

[105] Bhoi S.K., Kalita J., Misra U.K. (2012). Metabolic syndrome and insulin resistance in migraine. J. Headache Pain.

[106] Ali M., Hussein M., Magdy R., Khamis A., Al-Azayem S.A., Othman A.M., Ahmed A., Osama W. (2022). The potential impact of insulin resistance and metabolic syndrome on migraine headache characteristics. BMC Neurol..

[107] Zhang Q., Jin K., Chen B., Liu R., Cheng S., Zhang Y., Lu J. (2022). Overnutrition Induced Cognitive Impairment: Insulin Resistance, Gut-Brain Axis, and Neuroinflammation. Front. Neurosci..

[108] Van Dyken P., Lacoste B. (2018). Impact of Metabolic Syndrome on Neuroinflammation and the Blood-Brain Barrier. Front. Neurosci..

[109] Hagen K., Asvold B.O., Midthjell K., Stovner L.J., Zwart J.A., Linde M. (2018). Inverse relationship between type 1 diabetes mellitus and migraine. Data from the Nord-Trondelag Health Surveys 1995–1997 and 2006–2008. Cephalalgia.

[110] Berge L.I., Riise T., Fasmer O.B., Hundal O., Oedegaard K.J., Midthjell K., Lund A. (2013). Does diabetes have a protective effect on migraine?. Epidemiology.

[111] Aamodt A.H., Stovner L.J., Midthjell K., Hagen K., Zwart J.A. (2007). Headache prevalence related to diabetes mellitus. The Head-HUNT study. Eur. J. Neurol..

[112] Fagherazzi G., El Fatouhi D., Fournier A., Gusto G., Mancini F.R., Balkau B., Boutron-Ruault M.C., Kurth T., Bonnet F. (2019). Associations between Migraine and Type 2 Diabetes in Women: Findings From the E3N Cohort Study. JAMA Neurol..

[113] Friedman B.W., Mistry B., West J.R., Wollowitz A. (2014). The association between headache and elevated blood pressure among patients presenting to an ED. Am. J. Emerg. Med..

[114] Paolucci M., Altamura C., Vernieri F. (2021). The Role of Endothelial Dysfunction in the Pathophysiology and Cerebrovascular Effects of Migraine: A Narrative Review. J. Clin. Neurol..

[115] Tronvik E., Stovner L.J., Helde G., Sand T., Bovim G. (2003). Prophylactic treatment of migraine with an angiotensin II receptor blocker: A randomized controlled trial. JAMA.

[116] Mahmoud A.N., Mentias A., Elgendy A.Y., Qazi A., Barakat A.F., Saad M., Mohsen A., Abuzaid A., Mansoor H., Mojadidi M.K. (2018). Migraine and the risk of cardiovascular and cerebrovascular events: A meta-analysis of 16 cohort studies including 1 152 407 subjects. BMJ Open.

[117] Olesen J. (2008). The role of nitric oxide (NO) in migraine, tension-type headache and cluster headache. Pharmacol. Ther..

[118] Entonen A.H., Suominen S.B., Sillanmaki L.H., Rautava P.T., Kauniskangas K., Mantyselka P.T., Sumanen M., Koskenvuo M.J. (2022). Prevalent migraine as a predictor of incident hypertension. Eur. J. Public Health.

[119] Gales B.J., Bailey E.K., Reed A.N., Gales M.A. (2010). Angiotensin-converting enzyme inhibitors and angiotensin receptor blockers for the prevention of migraines. Ann. Pharmacother..

[120] Barbanti P., Aurilia C., Egeo G., Fofi L. (2010). Hypertension as a risk factor for migraine chronification. Neurol. Sci..

[121] Manna P., Jain S.K. (2015). Obesity, Oxidative Stress, Adipose Tissue Dysfunction, and the Associated Health Risks: Causes and Therapeutic Strategies. Metab. Syndr. Relat. Disord..

[122] Bleich S., Cutler D., Murray C., Adams A. (2008). Why is the developed world obese?. Annu. Rev. Public Health.

[123] Gelaye B., Sacco S., Brown W.J., Nitchie H.L., Ornello R., Peterlin B.L. (2017). Body composition status and the risk of migraine: A meta-analysis. Neurology.

[124] Arzani M., Jahromi S.R., Ghorbani Z., Vahabizad F., Martelletti P., Ghaemi A., Sacco S., Togha M., School of Advanced Studies of the European Headache Federation (EHF-SAS) (2020). Gut-brain Axis and migraine headache: A comprehensive review. J. Headache Pain.

[125] Westgate C.S.J., Israelsen I.M.E., Jensen R.H., Eftekhari S. (2021). Understanding the link between obesity and headache- with focus on migraine and idiopathic intracranial hypertension. J. Headache Pain.

[126] Kany S., Vollrath J.T., Relja B. (2019). Cytokines in Inflammatory Disease. Int. J. Mol. Sci..

[127] Wu X., Wang M., Li S., Zhang Y. (2016). Migraine and breast cancer risk: A meta-analysis of observational studies based on MOOSE compliant. Medicine.

[128] Elser H., Skajaa N., Ehrenstein V., Fuglsang C.H., Farkas D.K., Sorensen H.T. (2022). Cancer risk in patients with migraine: A population-based cohort study in Denmark. Headache.

[129] Chen C.H., Sheu J.J., Lin Y.C., Lin H.C. (2018). Association of migraines with brain tumors: A nationwide population-based study. J. Headache Pain.

[130] Goldlust S.A., Graber J.J., Bossert D.F., Avila E.K. (2010). Headache in patients with cancer. Curr. Pain Headache Rep..

[131] Bernstein C.A. (2021). Evaluation of headache in patients with cancer. Cancer.

[132] Weng S.C., Wu C.L., Kor C.T., Chiu P.F., Wu M.J., Chang C.C., Tarng D.C. (2017). Migraine and subsequent chronic kidney disease risk: A nationwide population-based cohort study. BMJ Open.

[133] Zhang W., Zhang L., Yang L., Xiao C., Wu X., Yan P., Cui H., Yang C., Zhu J., Wu X. (2023). Migraine, chronic kidney disease and kidney function: Observational and genetic analyses. Hum. Genet..

[134] Rahman A., Segasothy M., Samad S.A., Zulfiqar A., Rani M. (1993). Analgesic use and chronic renal disease in patients with headache. Headache.

[135] Arnold M. (2018). Headache Classification Committee of the International Headache Society (IHS) The International Classification of Headache Disorders, 3rd edition. Cephalalgia.

[136] Chhaya K.T., Mankad S., Shah M.K., Patel M., Desai D., Desai S.D. (2022). Headache Associated with Hemodialysis in Patients with End-Stage Renal Disease in India: A Common Yet Overlooked Comorbidity. Ann. Indian Acad. Neurol..

[137] Silberstein S.D. (2015). Preventive Migraine Treatment. Continuum.

[138] Kesserwani H. (2021). Migraine Triggers: An Overview of the Pharmacology, Biochemistry, Atmospherics, and Their Effects on Neural Networks. Cureus.

[139] Gazerani P. (2020). Migraine and Diet. Nutrients.

[140] Bagdy G., Riba P., Kecskemeti V., Chase D., Juhasz G. (2010). Headache-type adverse effects of NO donors: Vasodilation and beyond. Br. J. Pharmacol..

[141] Lacatusu C.M., Grigorescu E.D., Floria M., Onofriescu A., Mihai B.M. (2019). The Mediterranean Diet: From an Environment-Driven Food Culture to an Emerging Medical Prescription. Int. J. Environ. Res. Public Health.

[142] Aridi Y.S., Walker J.L., Roura E., Wright O.R.L. (2020). Adherence to the Mediterranean Diet and Chronic Disease in Australia: National Nutrition and Physical Activity Survey Analysis. Nutrients.

[143] Arab A., Khorvash F., Karimi E., Hadi A., Askari G. (2023). Associations between adherence to Mediterranean dietary pattern and frequency, duration, and severity of migraine headache: A cross-sectional study. Nutr. Neurosci..

[144] Sofi F., Abbate R., Gensini G.F., Casini A. (2010). Accruing evidence on benefits of adherence to the Mediterranean diet on health: An updated systematic review and meta-analysis. Am. J. Clin. Nutr..

[145] Alcalay R.N., Gu Y., Mejia-Santana H., Cote L., Marder K.S., Scarmeas N. (2012). The association between Mediterranean diet adherence and Parkinson’s disease. Mov. Disord..

[146] Liperoti R., Landi F., Fusco O., Bernabei R., Onder G. (2009). Omega-3 polyunsaturated fatty acids and depression: A review of the evidence. Curr. Pharm. Des..

[147] Liao Y., Xie B., Zhang H., He Q., Guo L., Subramanieapillai M., Fan B., Lu C., McIntyre R.S. (2019). Efficacy of omega-3 PUFAs in depression: A meta-analysis. Transl. Psychiatry.

[148] Tamir H., Mahadik S.P., Rapport M.M. (1976). Fractionation of synaptic membranes with sodium diatrizoate. Anal. Biochem..

[149] Azzini E., Polito A., Fumagalli A., Intorre F., Venneria E., Durazzo A., Zaccaria M., Ciarapica D., Foddai M.S., Mauro B. (2011). Mediterranean Diet Effect: An Italian picture. Nutr. J..

[150] Bakirhan H., Yildiran H., Uyar Cankay T. (2022). Associations between diet quality, DASH and Mediterranean dietary patterns and migraine characteristics. Nutr. Neurosci..

[151] Ghoreishy S.M., Askari G., Mohammadi H., Campbell M.S., Khorvash F., Arab A. (2022). Associations between potential inflammatory properties of the diet and frequency, duration, and severity of migraine headaches: A cross-sectional study. Sci. Rep..

[152] Martins L.B., Braga Tibaes J.R., Dos Santos Rodrigues A.M., Hassanzadeh Keshteli A., Karam Vono C., Borges E.B.J., Horta P.M., Teixeira A.L., Matos Ferreira A.V. (2022). The quality and inflammatory index of the diet of patients with migraine. Nutr. Neurosci..

[153] Merra G., Noce A., Marrone G., Cintoni M., Tarsitano M.G., Capacci A., De Lorenzo A. (2020). Influence of Mediterranean Diet on Human Gut Microbiota. Nutrients.

[154] D’Andrea Meira I., Romao T.T., Pires do Prado H.J., Kruger L.T., Pires M.E.P., da Conceicao P.O. (2019). Ketogenic Diet and Epilepsy: What We Know So Far. Front. Neurosci..

[155] Longo R., Peri C., Cricri D., Coppi L., Caruso D., Mitro N., De Fabiani E., Crestani M. (2019). Ketogenic Diet: A New Light Shining on Old but Gold Biochemistry. Nutrients.

[156] Hall K.D., Chen K.Y., Guo J., Lam Y.Y., Leibel R.L., Mayer L.E., Reitman M.L., Rosenbaum M., Smith S.R., Walsh B.T. (2016). Energy expenditure and body composition changes after an isocaloric ketogenic diet in overweight and obese men. Am. J. Clin. Nutr..

[157] Koh S., Dupuis N., Auvin S. (2020). Ketogenic diet and Neuroinflammation. Epilepsy Res..

[158] Greco T., Glenn T.C., Hovda D.A., Prins M.L. (2016). Ketogenic diet decreases oxidative stress and improves mitochondrial respiratory complex activity. J. Cereb. Blood Flow. Metab..

[159] Paoli A., Mancin L., Bianco A., Thomas E., Mota J.F., Piccini F. (2019). Ketogenic Diet and Microbiota: Friends or Enemies?. Genes.

[160] Borkum J.M. (2016). Migraine Triggers and Oxidative Stress: A Narrative Review and Synthesis. Headache.

[161] Anderson G. (2019). Integrating Pathophysiology in Migraine: Role of the Gut Microbiome and Melatonin. Curr. Pharm. Des..

[162] Di Lorenzo C., Ballerini G., Barbanti P., Bernardini A., D’Arrigo G., Egeo G., Frediani F., Garbo R., Pierangeli G., Prudenzano M.P. (2021). Applications of Ketogenic Diets in Patients with Headache: Clinical Recommendations. Nutrients.

[163] Kechagia M., Basoulis D., Konstantopoulou S., Dimitriadi D., Gyftopoulou K., Skarmoutsou N., Fakiri E.M. (2013). Health benefits of probiotics: A review. ISRN Nutr..

[164] Fijan S. (2014). Microorganisms with claimed probiotic properties: An overview of recent literature. Int. J. Environ. Res. Public Health.

[165] Vlasova A.N., Kandasamy S., Chattha K.S., Rajashekara G., Saif L.J. (2016). Comparison of probiotic lactobacilli and bifidobacteria effects, immune responses and rotavirus vaccines and infection in different host species. Vet. Immunol. Immunopathol..

[166] Naghibi M.M., Day R., Stone S., Harper A. (2019). Probiotics for the Prophylaxis of Migraine: A Systematic Review of Randomized Placebo Controlled Trials. J. Clin. Med..

[167] Caetano M.A.F., Castelucci P. (2022). Role of short chain fatty acids in gut health and possible therapeutic approaches in inflammatory bowel diseases. World J. Clin. Cases.

[168] van Hemert S., Breedveld A.C., Rovers J.M., Vermeiden J.P., Witteman B.J., Smits M.G., de Roos N.M. (2014). Migraine associated with gastrointestinal disorders: Review of the literature and clinical implications. Front. Neurol..

[169] Hemarajata P., Versalovic J. (2013). Effects of probiotics on gut microbiota: Mechanisms of intestinal immunomodulation and neuromodulation. Therap. Adv. Gastroenterol..

[170] Dai Y.J., Wang H.Y., Wang X.J., Kaye A.D., Sun Y.H. (2017). Potential Beneficial Effects of Probiotics on Human Migraine Headache: A Literature Review. Pain Physician.

[171] Bedu-Ferrari C., Biscarrat P., Langella P., Cherbuy C. (2022). Prebiotics and the Human Gut Microbiota: From Breakdown Mechanisms to the Impact on Metabolic Health. Nutrients.

[172] Macfarlane G.T., Steed H., Macfarlane S. (2008). Bacterial metabolism and health-related effects of galacto-oligosaccharides and other prebiotics. J. Appl. Microbiol..

[173] Divyashri G., Sadanandan B., Chidambara Murthy K.N., Shetty K., Mamta K. (2021). Neuroprotective Potential of Non-Digestible Oligosaccharides: An Overview of Experimental Evidence. Front. Pharmacol..

[174] Levine E.S., Crozier R.A., Black I.B., Plummer M.R. (1998). Brain-derived neurotrophic factor modulates hippocampal synaptic transmission by increasing N-methyl-D-aspartic acid receptor activity. Proc. Natl. Acad. Sci. USA.

[175] Bull F.C., Al-Ansari S.S., Biddle S., Borodulin K., Buman M.P., Cardon G., Carty C., Chaput J.P., Chastin S., Chou R. (2020). World Health Organization 2020 guidelines on physical activity and sedentary behaviour. Br. J. Sports Med..

[176] Warburton D.E., Nicol C.W., Bredin S.S. (2006). Health benefits of physical activity: The evidence. Can. Med. Assoc. J..

[177] Cerda B., Perez M., Perez-Santiago J.D., Tornero-Aguilera J.F., Gonzalez-Soltero R., Larrosa M. (2016). Gut Microbiota Modification: Another Piece in the Puzzle of the Benefits of Physical Exercise in Health?. Front. Physiol..

[178] Zhang L., Liu Y., Wang X., Zhang X. (2023). Physical Exercise and Diet: Regulation of Gut Microbiota to Prevent and Treat Metabolic Disorders to Maintain Health. Nutrients.

[179] Campaniello D., Corbo M.R., Sinigaglia M., Speranza B., Racioppo A., Altieri C., Bevilacqua A. (2022). How Diet and Physical Activity Modulate Gut Microbiota: Evidence, and Perspectives. Nutrients.

[180] Rodrigues S., Silva J.M.D., Oliveira M.C.C., Santana C.M.F., Carvalho K.M., Barbosa B. (2021). Physical exercise as a non-pharmacological strategy for reducing behavioral and psychological symptoms in elderly with mild cognitive impairment and dementia: A systematic review of randomized clinical trials. Arq. Neuropsiquiatr..

[181] Mizutani T., Ishizaka A., Koga M., Tsutsumi T., Yotsuyanagi H. (2022). Role of Microbiota in Viral Infections and Pathological Progression. Viruses.

[182] Cataldi S., Bonavolonta V., Poli L., Clemente F.M., De Candia M., Carvutto R., Silva A.F., Badicu G., Greco G., Fischetti F. (2022). The Relationship between Physical Activity, Physical Exercise, and Human Gut Microbiota in Healthy and Unhealthy Subjects: A Systematic Review. Biology.

[183] Song T.J., Chu M.K. (2021). Exercise in Treatment of Migraine Including Chronic Migraine. Curr. Pain Headache Rep..

[184] Lippi G., Mattiuzzi C., Sanchis-Gomar F. (2018). Physical exercise and migraine: For or against?. Ann. Transl. Med..

[185] Song T.J., Chu M.K., Sohn J.H., Ahn H.Y., Lee S.H., Cho S.J. (2018). Effect of Vitamin D Deficiency on the Frequency of Headaches in Migraine. J. Clin. Neurol..

[186] Eyles D.W. (2021). Vitamin D: Brain and Behavior. JBMR Plus.

[187] Das S., Hasan M.M., Mohsin M., Jeorge D.H., Rasul M.G., Khan A.R., Gazi M.A., Ahmed T. (2022). Sunlight, dietary habits, genetic polymorphisms and vitamin D deficiency in urban and rural infants of Bangladesh. Sci. Rep..

[188] Amiri P., Kazeminasab S., Nejadghaderi S.A., Mohammadinasab R., Pourfathi H., Araj-Khodaei M., Sullman M.J.M., Kolahi A.A., Safiri S. (2021). Migraine: A Review on Its History, Global Epidemiology, Risk Factors, and Comorbidities. Front. Neurol..

[189] Niu P.P., Wang X., Xu Y.M. (2022). Higher Circulating Vitamin D Levels Are Associated With Decreased Migraine Risk: A Mendelian Randomization Study. Front. Nutr..

[190] Zandifar A., Masjedi S.S., Banihashemi M., Asgari F., Manouchehri N., Ebrahimi H., Haghdoost F., Saadatnia M. (2014). Vitamin D status in migraine patients: A case-control study. Biomed. Res. Int..

[191] Togha M., Razeghi Jahromi S., Ghorbani Z., Martami F., Seifishahpar M. (2018). Serum Vitamin D Status in a Group of Migraine Patients Compared With Healthy Controls: A Case-Control Study. Headache.

[192] Acharya U., Gau J.T., Horvath W., Ventura P., Hsueh C.T., Carlsen W. (2008). Hemolysis and hyperhomocysteinemia caused by cobalamin deficiency: Three case reports and review of the literature. J. Hematol. Oncol..

[193] Ustun Ozek S. (2022). A study on the correlation between pain frequency and severity and vitamin B12 levels in episodic and chronic migraine. Arq. Neuropsiquiatr..

[194] D’Onofrio F., Raimo S., Spitaleri D., Casucci G., Bussone G. (2017). Usefulness of nutraceuticals in migraine prophylaxis. Neurol. Sci..

[195] Faraji H., Paknahad Z., Chitsaz A. (2018). Dietary Intake of Thiamine in Migraine Patients and Healthy Subjects: A Case-Control Study. Clin. Nutr. Res..

[196] Fila M., Chojnacki C., Chojnacki J., Blasiak J. (2021). Nutrients to Improve Mitochondrial Function to Reduce Brain Energy Deficit and Oxidative Stress in Migraine. Nutrients.

[197] Maresz K. (2021). Growing Evidence of a Proven Mechanism Shows Vitamin K2 Can Impact Health Conditions Beyond Bone and Cardiovascular. Integr. Med..

[198] Abbaspour N., Hurrell R., Kelishadi R. (2014). Review on iron and its importance for human health. J. Res. Med. Sci..

[199] Meng S.H., Zhou H.B., Li X., Wang M.X., Kang L.X., Fu J.M., Li X., Li X.T., Zhao Y.S. (2021). Association between Dietary Iron Intake and Serum Ferritin and Severe Headache or Migraine. Front. Nutr..

[200] Torres-Ferrus M., Ursitti F., Alpuente A., Brunello F., Chiappino D., de Vries T., Di Marco S., Ferlisi S., Guerritore L., Gonzalez-Garcia N. (2020). From transformation to chronification of migraine: Pathophysiological and clinical aspects. J. Headache Pain.

[201] Liu H., Wang D., Wu F., Dong Z., Yu S. (2023). Association between inflammatory potential of diet and self-reported severe headache or migraine: A cross-sectional study of the National Health and Nutrition Examination Survey. Nutrition.

[202] Pamuk G.E., Top M.S., Uyanik M.S., Koker H., Akker M., Ak R., Yurekli O.A., Celik Y. (2016). Is iron-deficiency anemia associated with migraine? Is there a role for anxiety and depression?. Wien. Klin. Wochenschr..

[203] Singla R.K., Dubey A.K., Garg A., Sharma R.K., Fiorino M., Ameen S.M., Haddad M.A., Al-Hiary M. (2019). Natural Polyphenols: Chemical Classification, Definition of Classes, Subcategories, and Structures. J. AOAC Int..

[204] Bakirhan H., Pehlivan M., Uyar Cankay T., Kocak M. (2022). Migraine severity, disability, and duration: Is a good diet quality, high intake of phytochemicals and polyphenols important?. Front. Nutr..

[205] Goschorska M., Gutowska I., Baranowska-Bosiacka I., Barczak K., Chlubek D. (2020). The Use of Antioxidants in the Treatment of Migraine. Antioxidants.

[206] Nowaczewska M., Wicinski M., Kazmierczak W., Kazmierczak H. (2020). To Eat or Not to Eat: A Review of the Relationship between Chocolate and Migraines. Nutrients.

[207] Meng S.H., Wang M.X., Kang L.X., Fu J.M., Zhou H.B., Li X., Li X., Li X.T., Zhao Y.S. (2021). Dietary Intake of Calcium and Magnesium in Relation to Severe Headache or Migraine. Front. Nutr..

[208] Domitrz I., Cegielska J. (2022). Magnesium as an Important Factor in the Pathogenesis and Treatment of Migraine-From Theory to Practice. Nutrients.

[209] Yamanaka R., Shindo Y., Oka K. (2019). Magnesium Is a Key Player in Neuronal Maturation and Neuropathology. Int. J. Mol. Sci..

[210] Li K., Wang X.F., Li D.Y., Chen Y.C., Zhao L.J., Liu X.G., Guo Y.F., Shen J., Lin X., Deng J. (2018). The good, the bad, and the ugly of calcium supplementation: A review of calcium intake on human health. Clin. Interv. Aging.

[211] Schnedl W.J., Enko D. (2021). Histamine Intolerance Originates in the Gut. Nutrients.

[212] Branco A., Yoshikawa F.S.Y., Pietrobon A.J., Sato M.N. (2018). Role of Histamine in Modulating the Immune Response and Inflammation. Mediators Inflamm..

[213] Costa F., Beltrami E., Mellone S., Sacchetti S., Boggio E., Gigliotti C.L., Stoppa I., Dianzani U., Rolla R., Giordano M. (2023). Genes and Microbiota Interaction in Monogenic Autoimmune Disorders. Biomedicines.

[214] Durak-Dados A., Michalski M., Osek J. (2020). Histamine and Other Biogenic Amines in Food. J. Vet. Res..

[215] Liu X., Yu Y., Hou L., Yu Y., Wu Y., Wu S., He Y., Ge Y., Wei Y., Luo Q. (2023). Association between dietary habits and the risk of migraine: A Mendelian randomization study. Front. Nutr..

[216] Ruiz-Capillas C., Herrero A.M. (2019). Impact of Biogenic Amines on Food Quality and Safety. Foods.

[217] Izquierdo-Casas J., Comas-Baste O., Latorre-Moratalla M.L., Lorente-Gascon M., Duelo A., Soler-Singla L., Vidal-Carou M.C. (2019). Diamine oxidase (DAO) supplement reduces headache in episodic migraine patients with DAO deficiency: A randomized double-blind trial. Clin. Nutr..

[218] Okutan G., Ruiz Casares E., Perucho Alcalde T., Sanchez Nino G.M., Penades B.F., Terren Lora A., Torrente Estringana L., Lopez Oliva S., San Mauro Martin I. (2023). Prevalence of Genetic Diamine Oxidase (DAO) Deficiency in Female Patients with Fibromyalgia in Spain. Biomedicines.

[219] Brown R.W., Chadwick D.R., Bott T., West H.M., Wilson P., Hodgins G.R., Snape C.E., Jones D.L. (2023). Biochar application to temperate grasslands: Challenges and opportunities for delivering multiple ecosystem services. Biochar.

[220] Esposito F., Montuori P., Schettino M., Velotto S., Stasi T., Romano R., Cirillo T. (2019). Level of Biogenic Amines in Red and White Wines, Dietary Exposure, and Histamine-Mediated Symptoms upon Wine Ingestion. Molecules.

